# Waldenström Macroglobulinemia–Induced Cardiac Amyloid Light Chain Amyloidosis

**DOI:** 10.31486/toj.23.0144

**Published:** 2024

**Authors:** Jerry Fan, Michael Chukwu, Corry B. Sanford, Deborah Jebakumar, Nicholas Quitoriano, Vinh Nguyen

**Affiliations:** ^1^ Division of Cardiology, Baylor Scott & White Medical Center, Temple, TX; ^2^ Department of Internal Medicine, Baylor Scott & White Medical Center, Temple, TX; ^3^ Department of Pathology, Baylor Scott & White Medical Center, Temple, TX

**Keywords:** *Amyloidosis*, *cardiomyopathies*, *Waldenstrom macroglobulinemia*

## Abstract

**Background:** Waldenström macroglobulinemia is a rare cancer of plasma cells characterized by the excessive production of immunoglobulin M (IgM). IgM-associated systemic amyloid light chain (AL) amyloidosis is a rare complication of Waldenström macroglobulinemia, characterized by the misfolding of lambda light chains that deposit in various organs, including the heart. We describe a case of progressive nonischemic cardiomyopathy secondary to Waldenström macroglobulinemia and IgM-associated AL amyloidosis that was refractory to medical therapy and highlight the challenges in diagnosis and management.

**Case Report:** A 64-year-old male with hypertension presented with symptoms of heart failure. Diagnostic workup revealed evidence of Waldenström macroglobulinemia and IgM-associated systemic AL amyloidosis affecting the heart. Further investigations confirmed the presence of Waldenström macroglobulinemia with lambda-restricted lymphoplasmacytic infiltrate in the bone marrow. Renal biopsy revealed amyloid nephropathy, and endomyocardial biopsy showed extensive deposits of fibrillary material consistent with cardiac amyloidosis. Because of the patient's advanced disease state and frailty, the decision was made to focus on comfort care with hospice.

**Conclusion:** Waldenström macroglobulinemia–induced cardiac AL amyloidosis is a challenging clinical scenario characterized by the coexistence of 2 distinct hematologic disorders impacting cardiac function. Diagnosis requires a comprehensive evaluation, and management necessitates a multidisciplinary approach targeting both Waldenström macroglobulinemia and cardiac amyloidosis. Further research and collaboration are needed to improve diagnostic techniques, refine treatment approaches, and enhance patient outcomes for this rare and complex condition.

## INTRODUCTION

Waldenström macroglobulinemia, a rare cancer of the plasma cells, is characterized by the excessive production of immunoglobulin M (IgM), resulting in monoclonal gammopathy.^1^ Waldenström macroglobulinemia is an indolent disease that, depending on the presentation, may not require treatment; however, it can lead to the accumulation of monoclonal proteins and is associated with various clinical manifestations such as anemia, compromised immune function, bleeding diathesis, and nervous system pathology.^1,2^ A particularly uncommon complication of Waldenström macroglobulinemia is the development of IgM-associated systemic amyloid light chain (AL) amyloidosis, characterized by the misfolding of lambda light chains that deposit in various organs.^1,3^

Patients with Waldenström macroglobulinemia–induced AL amyloidosis often present with symptoms mimicking other common conditions, leading to delays in diagnosis and initiation of appropriate therapy.^1^ The deposition of amyloid fibrils in the heart tissue disrupts the heart's normal structure and function, leading to restrictive cardiomyopathy, heart failure, arrhythmias, and conduction abnormalities.^1,4,5^ Given the complexity and rarity of Waldenström macroglobulinemia–induced AL amyloidosis, a multidisciplinary approach involving hematologists, cardiologists, and other specialists is essential for optimal management. We describe a case of progressive nonischemic cardiomyopathy from Waldenström macroglobulinemia–induced AL amyloidosis that was refractory to medical therapy. The case underscores the challenges in diagnosis and management and the urgent need for continued research to improve outcomes in patients with this rare and complex condition.

## CASE REPORT

A 64-year-old male with hypertension presented with a 2-year onset of fatigue, peripheral edema, exertional dyspnea, and frailty. Physical examination revealed bibasilar crackles and 3+ pitting edema in both lower extremities. Electrocardiogram showed left atrial enlargement and left ventricular hypertrophy. Further investigations were notable for elevated B-type natriuretic peptide of 4,135 pg/mL (reference value, ≤100 pg/mL) and troponin 1 of 0.60 ng/mL (reference range, 0.00-0.09 ng/mL). Transthoracic echocardiogram showed reduced systolic function (left ventricular ejection fraction 36%-40%), diastolic dysfunction, and apical preservation of global longitudinal strain. Serum protein electrophoresis revealed elevated IgM of 2,951 mg/dL (reference range, 40-230 mg/dL), kappa free light chains of 67.13 mg/L (reference range, 3.30-19.40 mg/L), and lambda free light chains of 425.58 mg/L (reference range, 5.71-26.30 mg/L), with a decreased kappa/lambda ratio of 0.16 (reference range, 0.26-1.65). Immunofixation confirmed the presence of a monoclonal protein band. Urine protein/creatinine ratio was elevated at 6.73 (reference range, 0.00-0.19).

**Figure 1.**
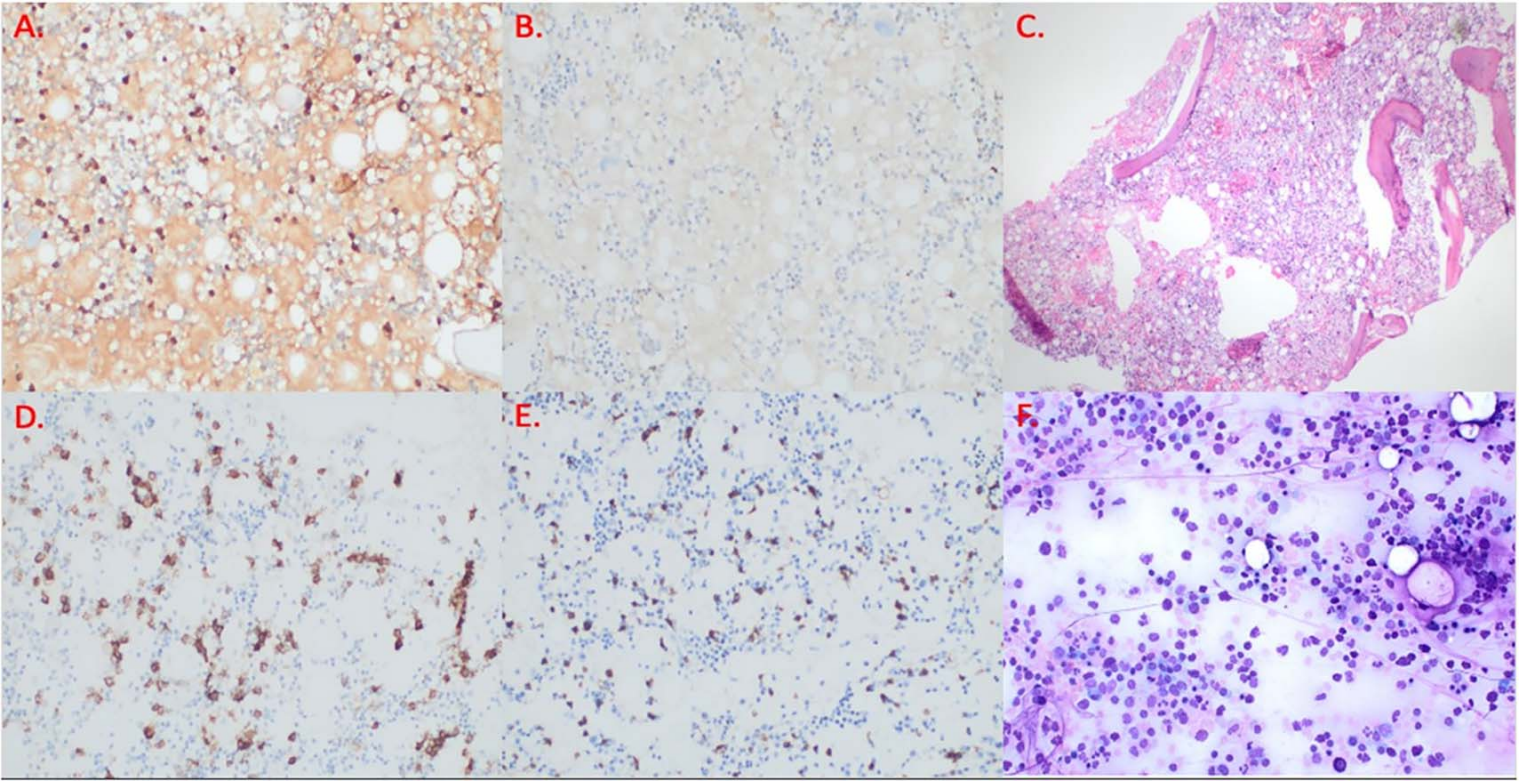
Bone marrow biopsy shows (A, B) lambda-restricted plasma cells with approximately 15% bone marrow cellularity (magnification ×200), (C) bone marrow cellularity at 30% to 40% (magnification ×40), and (D, E) plasma cell population via CD138 and CD20 (magnification ×200). (F) Aspirate of bone marrow shows slight increase in plasma cells and mature lymphocytes (magnification ×200).

Bone marrow biopsy confirmed Waldenström macroglobulinemia, with a lambda-restricted lymphoplasmacytic infiltrate (CD5/CD10 negative) accounting for approximately 15% of marrow cellularity (Figure 1). The aspirate had negative Congo red staining and normal cytogenetics but showed an alteration in MYD88 L265P, a prevalent somatic mutation in patients with Waldenström macroglobulinemia and IgM monoclonal gammopathy of undetermined significance.

**Figure 2.**
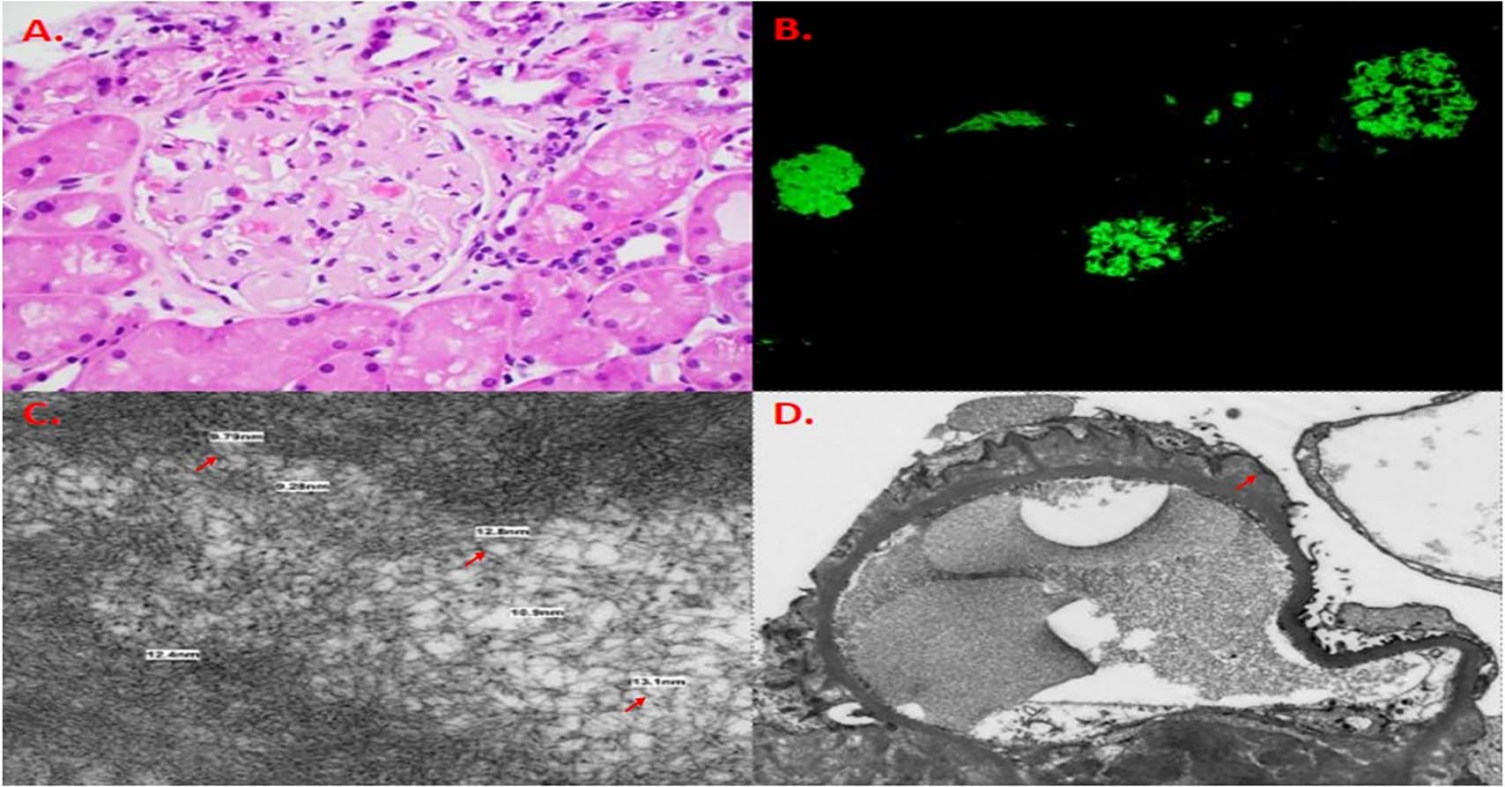
Renal biopsy shows amyloid nephropathy: (A) glomerulus with nodularity and prominent mesangial expansion by amorphous, eosinophilic, and acellular material; and (B) glomerulus with intense thioflavin T–positive immunofluorescent staining. Electron microscopy shows (C) nonbranching, randomly disposed small-diameter fibrils (arrows) and (D) fibrillary deposits in the mesangial matrix and along the capillary loops (arrow).

**Figure 3.**
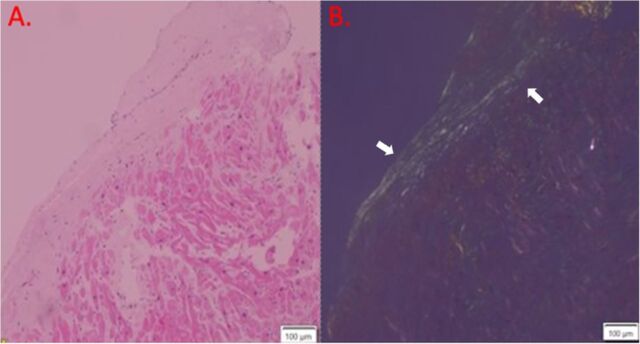
Endomyocardial biopsy shows myocardial amyloid fibril deposition. (A) Normal hematoxylin and eosin stain of the endomyocardium. (B) Congo red staining of the endomyocardium shows apple-green birefringence under polarized light (arrows).

Renal biopsy revealed amyloid nephropathy with nodularity and prominent mesangial expansion by amorphous, eosinophilic, and acellular material, as well as thioflavin T–positive nonbranching fibrils (Figure 2). The endomyocardial biopsy showed extensive deposits of fibrillary material and positive Congo red staining consistent with cardiac amyloidosis (Figure 3). Typing from the biopsies confirmed AL (lambda) amyloid.

The patient's disease course was complicated by renal failure requiring hemodialysis. Because of the patient's advanced disease state and frailty, the decision was made to focus on comfort care with hospice after a 22-day hospitalization. Three days after discharge, the patient succumbed to his disease and died.

## DISCUSSION

Waldenström macroglobulinemia is an uncommon and slow-growing cancer of plasma cells in which malignant plasma cells in the bone marrow produce an excessive amount of IgM.^1^ Patients may develop a rare complication of this condition known as Waldenström macroglobulinemia–induced AL amyloidosis, characterized by misfolding of lambda light chains that deposit in various organs due to mutations in MYD88 and CXCR4.^1,4,6^ Concurrent Waldenström macroglobulinemia and AL amyloidosis occur in approximately 7.5% of patients with Waldenström macroglobulinemia.^7^ The fibrils can deposit in multiple organs, including heart tissue where they can disrupt normal myocardial structure and function.^1^

While Waldenström macroglobulinemia usually has a relatively benign course, patients with a concurrent diagnosis of AL amyloidosis require prompt treatment.^8^ High suspicion for Waldenström macroglobulinemia–induced AL amyloidosis is crucial because of the widely varied presentations that can often mimic other conditions.^1^ Cardiac involvement can mimic heart failure symptoms with shortness of breath, peripheral edema, and fatigue, and peripheral involvement can mimic diabetic neuropathy symptoms with numbness, tingling, and neuropathy in the extremities.^1^

Because of the varied presentation, diagnosis is often delayed. In a cohort of 49 patients, 10 (20%) patients were diagnosed simultaneously with Waldenström macroglobulinemia and AL amyloidosis; AL amyloidosis was diagnosed a median of 3 months after Waldenström macroglobulinemia in 27 (56%) patients; and 12 (24%) patients were diagnosed with AL amyloidosis >5 years after the diagnosis of Waldenström macroglobulinemia.^4^ The damage often remains silent until end organ dysfunction is present. Good screening tests are the following biomarkers: 24-hour urinary albumin, urinary albumin/creatinine ratio, brain natriuretic peptide, creatinine, levels of IgM monoclonal proteins, and levels of free light chains.^1,6,7^ Abnormal levels should prompt additional screening measures such as echocardiography, bone marrow biopsy, and fat pad biopsy with Congo red staining.^1,8^

The management of combined Waldenström macroglobulinemia and AL amyloidosis requires a tailored approach that considers the dual pathology. Prospective clinical trials are lacking, with the majority of treatment options and response information from case studies and a consensus panel report from large academic centers.^2,7^

Waldenström macroglobulinemia does not always require treatment and can be monitored; however, if treatment is needed, Waldenström macroglobulinemia is usually treated with a combination of anti-CD20 antibody (rituximab), proteasome inhibitor (bortezomib), alkylating agent (chlorambucil or melphalan), and Bruton tyrosine kinase inhibitor (ibrutinib), while AL amyloidosis is usually treated with a combination of a proteasome inhibitor (bortezomib), cyclophosphamide, dexamethasone, and anti-CD38 antibody (daratumumab).^2,6,7^

For Waldenström macroglobulinemia–induced AL amyloidosis, effective therapy involves chemoimmunotherapy combinations rather than traditional alkylating regimens or single-agent rituximab.^3^

While the survival for each individual disease is relatively good—10+ years for Waldenström macroglobulinemia and 6 to 8 years for AL amyloidosis—the coexistence of both diseases portends a poor prognosis even with combination chemoimmunotherapy. The best results are seen in patients who qualify for autologous stem cell transplant, often not an option for patients of advanced age.^2,4,7^

## CONCLUSION

Waldenström macroglobulinemia–induced AL amyloidosis is a challenging clinical scenario characterized by the coexistence of 2 distinct hematologic disorders that impact cardiac function. Diagnosis requires a comprehensive evaluation, and management necessitates a multidisciplinary approach targeting both Waldenström macroglobulinemia and cardiac amyloidosis. Further research and collaboration are needed to improve diagnostic techniques, refine treatment approaches, and enhance patient outcomes for this rare and complex condition.

## Notes

*This article meets the Accreditation Council for Graduate Medical Education and the American Board of Medical Specialties Maintenance of Certification competencies for Patient Care and Medical Knowledge.*
